# Analysis of STAT1 Activation by Six FGFR3 Mutants Associated with Skeletal Dysplasia Undermines Dominant Role of STAT1 in FGFR3 Signaling in Cartilage

**DOI:** 10.1371/journal.pone.0003961

**Published:** 2008-12-17

**Authors:** Pavel Krejci, Lisa Salazar, Tamara A. Kashiwada, Katarina Chlebova, Alena Salasova, Leslie Michels Thompson, Vitezslav Bryja, Alois Kozubik, William R. Wilcox

**Affiliations:** 1 Department of Animal Physiology and Immunology, Institute of Experimental Biology, Masaryk University, Brno, Czech Republic; 2 Department of Cytokinetics, Institute of Biophysics ASCR, Brno, Czech Republic; 3 Medical Genetics Institute, Cedars-Sinai Medical Center, Los Angeles, California, United States of America; 4 Department of Psychiatry and Human Behavior, University of California Irvine, Irvine, California, United States of America; 5 Department of Pediatrics, University of California Los Angeles School of Medicine, Los Angeles, California, United States of America; Charité-Universitätsmedizin Berlin, Germany

## Abstract

Activating mutations in FGFR3 tyrosine kinase cause several forms of human skeletal dysplasia. Although the mechanisms of FGFR3 action in cartilage are not completely understood, it is believed that the STAT1 transcription factor plays a central role in pathogenic FGFR3 signaling. Here, we analyzed STAT1 activation by the N540K, G380R, R248C, Y373C, K650M and K650E-FGFR3 mutants associated with skeletal dysplasias. In a cell-free kinase assay, only K650M and K650E-FGFR3 caused activatory STAT1(Y701) phosphorylation. Similarly, in RCS chondrocytes, HeLa, and 293T cellular environments, only K650M and K650E-FGFR3 caused strong STAT1 activation. Other FGFR3 mutants caused weak (HeLa) or no activation (293T and RCS). This contrasted with ERK MAP kinase activation, which was strongly induced by all six mutants and correlated with the inhibition of proliferation in RCS chondrocytes. Thus the ability to activate STAT1 appears restricted to the K650M and K650E-FGFR3 mutants, which however account for only a small minority of the FGFR3-related skeletal dysplasia cases. Other pathways such as ERK should therefore be considered as central to pathological FGFR3 signaling in cartilage.

## Introduction

Activating mutations in FGFR3 receptor tyrosine kinase result in several forms of skeletal dysplasia. These range from the mild short-limbed dwarfism hypochondroplasia (HCH), to the most common genetic form of dwarfism achondroplasia (ACH), to severe achondroplasia with acanthosis nigricans and mental retardation (SADDAN), and to neonatal lethal thanatophoric dysplasia (TD) [1]. Despite recent progress in characterization of the mechanisms of FGFR3 signaling in cartilage, many aspects of this signaling remain unclear. At present, the FGFR3-mediated activation of STAT1 is believed to be a prominent mechanism of pathological FGFR3 signaling in cartilage [for reviews see references 2–4].

Several lines of evidence support this paradigm. First, the expression of FGFR3 harboring the highly activating TD-associated K650M or K650E mutations leads to activatory STAT1(Y701) phosphorylation in cells. This is evidenced in 293T embryonal kidney cells, PC12 pheochromocytoma cells, HeLa cervical cancer cells, and RCS chondrocytes [5]–[11]. This activation is accompanied with induction of differentiation in PC12 cells [9], or with STAT1 nuclear accumulation, increased levels of the p21^Waf1^ inhibitor of the cell cycle, and inhibition of proliferation in 293T cells [5]. Second, STAT1 accumulates and shows nuclear localization in the cartilage of TD-affected human fetuses as well as in mice carrying the K644E-FGFR3 mutation (homologous to human K650E) [12], [13]. Finally, two experimental studies show that the loss of STAT1 partially rescues the growth-inhibitory action of FGF signaling in chondrocytes [14], [15], both suggesting the role of STAT1 in the growth-inhibitory FGFR3 action in cartilage.

In contrast to the STAT1 hypothesis, several studies have demonstrated that ERK and p38 MAP kinases but not STAT1 are important for FGFR3-mediated growth inhibition of chondrocytes both *in vitro* and *in vivo* [11], [16]–[19].

The majority of evidence supporting the role of STAT1 in FGFR3 signaling in cartilage was obtained using the K650M or K650E mutants, which account for only a small subset of FGFR3-related skeletal dysplasia cases [1]. In addition, FGFR3 mutations only exaggerate the normal physiological function of FGFR3, which serves as a negative regulator of cartilage growth [2], [20]. No activation of STAT1 was found by wild-type FGFR3 in several studies [9]–[11], [21], [22], suggesting that STAT1 might not be the major intermediate in the FGFR3 signaling in cartilage. To answer this question, we compared six FGFR3 mutants, which together account for the majority of the known skeletal dysplasia cases, for their activation of STAT1 and ERK MAP kinase, and their effects on chondrocyte growth.

## Results and Discussion

### STAT1 activation by FGFR3 mutants in a cell-free kinase assay

**Figure 1:**
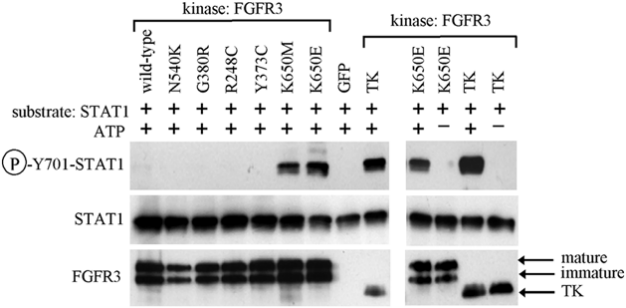
STAT1 activation by FGFR3 mutants in a cell-free kinase assay. Full-length wild-type FGFR3 or its activating mutants N540K, G380R, R248C, Y373C, K650M and K650E were expressed in CHO cells, activated by brief FGF2 treatment and purified by immunoprecipitation as described in Materials and Methods. Immunocomplexes were subjected to kinase assay with recombinant STAT1 as a substrate. Cells transfected with GFP vector serve as negative control for immunoprecipitation. Samples utilizing recombinant FGFR3 tyrosine kinase domain (TK) or those with omitted ATP serve as positive or negative control for kinase assay, respectively. Note that only K650M and K650E-FGFR3 mutants cause STAT1 phosphorylation, as evidenced by western blotting with antibody recognizing STAT1 only when phosphorylated at Y701 (P-Y701-STAT1). FGFR3 and STAT1 western blottings serve as controls for kinase or substrate quantity. Note the appearance of both immature and mature (glycosylated) FGFR3 forms expressed by CHO cells.

Although the expression of K650E-FGFR3 induces activatory STAT1(Y701) phosphorylation in cells [6], [8], the mechanism by which FGFR3 achieves this effect was not known until recently. Previously, we showed that K650E-FGFR3 interacts with STAT1 in cells and phosphorylates STAT1(Y701) in a cell-free kinase assay, thus serving as a STAT1 tyrosine kinase [11]. Here, we used the FGFR3 kinase assay to compare the ability of six different FGFR3 mutants associated with a range of skeletal dysplasia phenotypes to activate STAT1. Vectors carrying the wild-type FGFR3 as well as the N540K (HCH), G380R (ACH), R248C, Y373C, K650E (TD) and K650M (SADDAN and TD) mutants were expressed in CHO cells. Transfected cells were stimulated with FGF2 and FGFR3 was purified by immunoprecipitation 48 hours later. Subsequently, the FGFR3 immunocomplexes were subjected to a kinase assay with recombinant STAT1 as a substrate. In this experiment, only the K650M and K650E mutants induced activatory phosphorylation of STAT1(Y701); the other mutants as well as wild-type FGFR3 caused no detectable phosphorylation (Fig. 1).

The lack of STAT1 phosphorylation by the HCH and ACH mutants N540K and G380R as well as by the TD mutants R248C and Y373C might be explained by the lower levels of their activity when compared to K650E and K650M-FGFR3, although we do not favor this hypothesis. First, even the wild-type FGFR3 showed strong activity against its known substrate FRS2 under the same experimental conditions [23]. Second, R248C and Y373C are strongly activating mutants and cause TD similar to K650M and K650E, yet they did not phosphorylate STAT1 in a kinase assay (Fig. 1).

### STAT1 and ERK MAP kinase activation by FGFR3 mutants in HeLa, 293T and RCS cells

As the kinase assay is a simple, cell-free system where both kinase and substrate are in excess and there are no other proteins in the reaction, it might not entirely reflect *in vivo* situation. It is possible that N540K, G380R, R248C and Y373C mutants still activate STAT1 in cells, despite the lack of this capacity in a kinase assay (Fig. 1). For instance, other interacting proteins that facilitate FGFR3-mediated STAT1 activation could exist in cells. In addition, the lack of STAT1 phosphorylation by strongly *in vivo* activating TD mutations R248C and Y373C may stem from the fact that they affect the extracellular part of FGFR3 and activate FGFR3 by facilitating its dimerization [24]. Although we activated FGFR3 in CHO cells by exogenous FGF2 addition, it is unlikely that FGFR3 was immunoprecipitated in a form identical to intact cell membrane-associated FGF2/FGFR3 complexes. In contrast, K650M and K650E mutations affect the intracellular part of FGFR3 and activate FGFR3 via conformational changes that activate the kinase domain, which are normally initiated by ligand binding and autophosphorylation [25]. It is likely that this process is less sensitive to the potential artifacts introduced by the methods used, i.e. to the FGFR3 purification and kinase assay conditions.

Therefore, we hypothesized that the N540K, G380R, R248C and Y373C mutants might somehow phosphorylate STAT1(Y701) in cells similar to K650M and K650E. FGFR3 mutants were expressed in three different cellular environments (HeLa, 293T and RCS cells) and transfectants analyzed for STAT1(Y701) phosphorylation. HeLa and 293T cell lines were chosen since they were used previously to study the K650M or K650E-FGFR3-mediated STAT1 activation [5], [6], [8], [11], [21]; RCS chondrocytes were used since this cell line represents an established *in vitro* model for FGFR3 signaling in cartilage [14], [17], [18], [23], [26]–[29].

**Figure 2 pone-0003961-g002:**
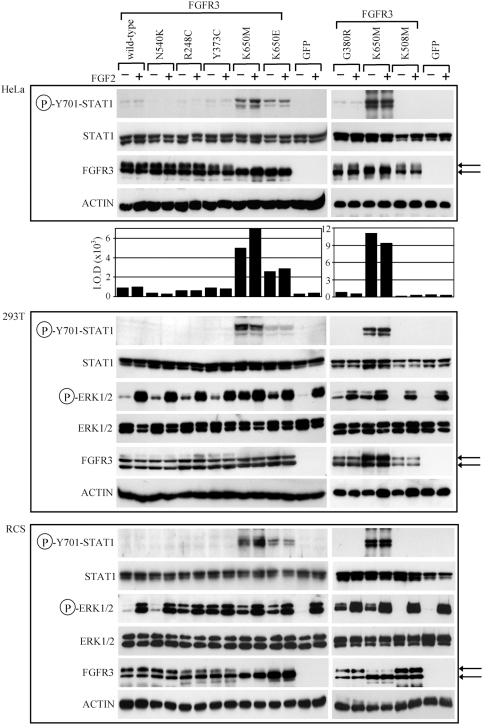
STAT1 and ERK1/2 activation by FGFR3 mutants in HeLa, 293T and RCS cells. HeLa, 293T and RCS cells, transfected with vectors expressing wild-type FGFR, activating FGFR3 mutants (N540K, G380R, R248C, Y373C, K650M and K650E), and kinase-inactive mutant K508M were grown for 48 hours, treated with FGF2 for 15 minutes and analyzed for given molecules by western blotting. The left and right panels represent independent experiments. Note that only K650M and K650E-FGFR3 cause strong activatory STAT1(Y701) phosphorylation whereas the other mutants cause weak (HeLa) or no activation (293T and RCS). This contrasts with ERK1/2 activation, which is induced by all six mutants in 293T and RCS cells (samples without FGF2 treatment). The P-Y701-STAT1 signal in HeLa cells was quantified by densitometry and graphed. Also note the differences in FGFR3 maturation, where HeLa and RCS cells but not 293T cells express mostly immature forms (lower arrow) of K650M and K650E-FGFR3. STAT1, ERK1/2 and ACTIN western blottings serve as loading controls. Cells transfected by GFP vector serve as negative control for transfection.

Cells were transfected with N540K, G380R, R248C, Y373C, K650M and K650E-FGFR3 mutants and analyzed for activatory STAT1(Y701) phosphorylation 48 hours later. Figure 2 shows that only the K650M and K650E mutants activated STAT1 in 293T and RCS cells, identically to the kinase assay data (Fig. 1). This phenotype was not affected by FGF2 treatment (20 ng/ml; 15 minutes). In contrast, we found a weak STAT1(Y701) activation in HeLa cells transfected with wild-type FGFR3 as well as with the G380R, R248C and Y373C mutants (Fig. 2). Although much lower than the activation induced by K650M or K650E mutants, it appeared to be dependent on the transgenic FGFR3-activity, since we found no STAT1(Y701) activation in cells transfected by the kinase-inactive K508M-FGFR3 mutant.

In cells transfected with FGFR3 mutants, we also monitored the recruitment of the ERK MAP kinase pathway, which is activated by FGFR3 signaling in cartilage in addition to STAT1 and regulates aberrant chondrocyte proliferation and/or differentiation in FGFR3-related skeletal dysplasias [16], [19], [30]. In 293T and RCS cells, all six FGFR3 mutants induced activatory ERK(T202/Y204) phosphorylation (Fig. 2). The activation of ERK by FGFR3 mutants could not be determined in HeLa cells due to high levels of endogenous active ERK in this cell line (data not shown). When compared to untreated cells, the levels of ERK activation are much higher in cells treated with FGF2 in all transfectants (Fig. 2). This is likely a result of FGF2-mediated activation of endogenous FGFR2/4 or FGFR3, expressed in 293T (data not shown) or RCS cells [18].

In summary, Figure 2 shows that ERK MAP kinase is activated by virtually all tested FGFR3 mutants in cells, including the weakly activating HCH and ACH mutants N540K and G380R, respectively. In contrast, STAT1 activation was restricted only to the K650M and K650E mutants in 293T and RCS cells. Our data are in agreement with others [8], [9], [21], who found no STAT1(Y701) phosphorylation by wild-type FGFR3 compared to K650M or K650E-FGFR3. In HeLa cells however, we found slight STAT1(Y701) phosphorylation induced by wild-type FGFR3 as well as G380R, R248C and Y373C mutants, similar to Legeai-Mallet *et al*. [31], Plowright *et al*. [32] and Ronchetti *et al*. [7], who found STAT1 activation in cells expressing R248C or Y373C-FGFR3. As determined by densitometry, the activation of STAT1 by wild-type FGFR3 in HeLa cells was ∼5.5-fold lower than in K650M (Fig. 2), similar to Harada et al. [10] or Su et al. [5], who found the wild-type FGFR3 activating STAT1 to the levels 4.8-fold or 20-fold lower than K650M.

Taken together, we found that wild-type FGFR3 as well as G380R, R248C and Y373C mutants may activate STAT1 depending on the cellular environment, although this activation is significantly lower when compared to K650M or K650E-FGFR3 (Fig. 2). How is this activation achieved? In the case of K650M and K650E mutants, the majority of STAT1 activation in cells is likely a result of direct phosphorylation and may result from intracellular activation [22]. For wild-type FGFR3 or G380R, R248C and Y373C mutants the direct FGFR3-mediated phosphorylation can not be ruled-out despite the lack of such activity in a kinase assay (Fig. 1). We speculate, however, that FGFR3 may facilitate STAT1 activation by its canonical pathways such as cytokine-JAK signaling [33]. Recently, we showed that STAT1 interacts with wild-type FGFR3 in cells and this interaction appears independent of FGFR3 activity since it is observed also for the K508M kinase-inactive mutant [11]. Thus the binding to transmembrane FGFR3 may recruit STAT1 to the membrane such that STAT1 is activated by JAK kinases.

### The effect of FGFR3 mutants on RCS chondrocyte proliferation in context of STAT1 and ERK MAP kinase activation

RCS chondrocytes represent the best characterized *in vitro* model to FGFR3-related skeletal dysplasia to date [17], [18], [23], [26]–[29]. RCS chondrocytes express wild-type FGFR3 and respond to its activation, via exogenously added FGF, by a potent growth arrest similar to the chondrocyte growth inhibition observed in cartilage of patients suffering from FGFR3-related skeletal dysplasias [17], [18], [26].

**Figure 3:**
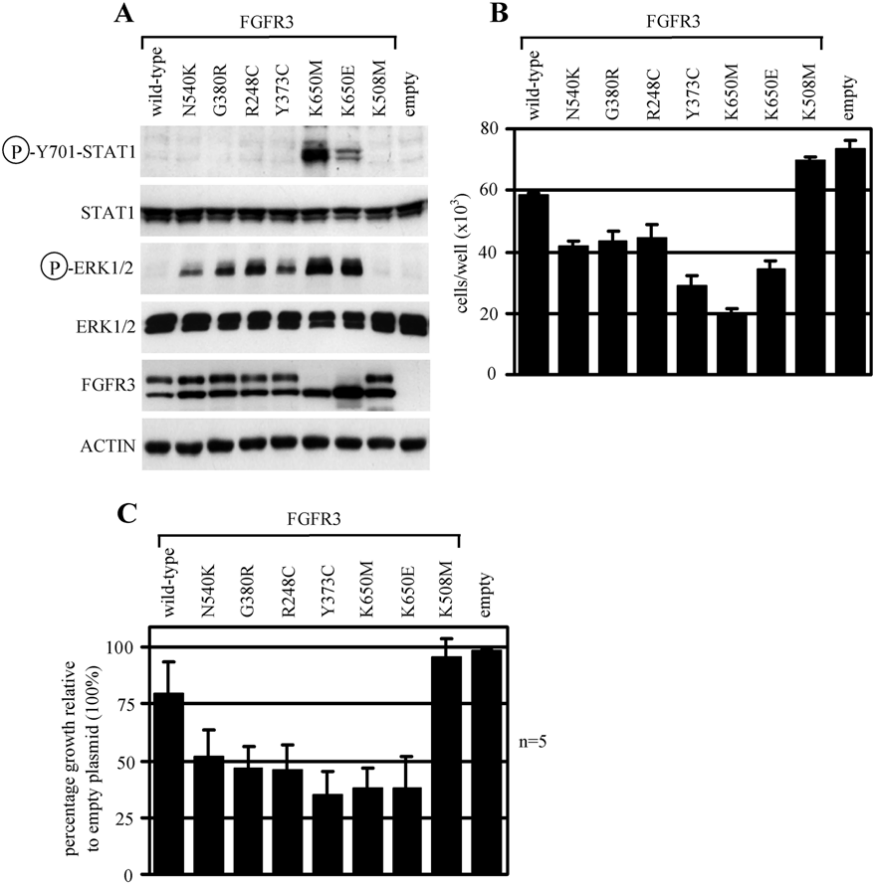
The effect of FGFR3 mutants on RCS chondrocyte proliferation in context of STAT1 and ERK1/2 activation. (A) RCS chondrocytes transfected with vectors expressing wild-type FGFR3, activating FGFR3 mutants (N540K, G380R, R248C, Y373C, K650E and K650M), and kinase-inactive mutant K508M were grown for 48 hours and analyzed for the indicated molecules by western blotting. Note differential STAT1 and ERK activation by the activating FGFR3 mutants. Cells transfected with empty plasmid (pcDNA3) serve as negative control for transfection. (B) RCS chondrocytes were transfected as described in (A), grown for 72 hours and counted. Note the inhibition of RCS growth by wild-type FGFR3 as well as the activating mutants, as compared to cells transfected either by kinase-inactive K508M-FGFR3 or an empty plasmid. The data represent an average from four individually transfected wells with indicated standard deviation. The cell count difference compared between cells transfected with wild-type FGFR3 and empty plasmid, as well as the cell count difference between cells transfected with wild-type FGFR3 and N540K, G380R, R248C, Y373C, K650M and K650E mutants, were statistically significant (Student's *t*-test, *p*<0.01). (C) The experiment shown on (B) was repeated five times to eliminate the variance associated with differential transfection efficiency. The differences in percentages of growth compared between cells transfected with wild-type FGFR3 and empty plasmid, and between cells transfected with wild-type FGFR3 and N540K, G380R, R248C, Y373C, K650M and K650E mutants, were statistically significant (Student's *t*-test, *p*<0.01).

We used RCS chondrocytes to evaluate the effect of FGFR3 mutants on cell proliferation in the context of STAT1 and ERK activation. As expected, 48 hour-long expression of N540K, G380R, R248C, Y373C, K650M and K650E-FGFR3 led to STAT1 activation only in case of K650M and K650E mutants in contrast to ERK activation, which was observed for all six mutants (Fig. 3A). For the growth arrest assay, RCS chondrocytes were transfected in 24-well plates, grown for 72 hours and counted. Figure 3B shows that all six FGFR3 mutants caused significant growth arrest when compared to cells transfected with wild-type FGFR3, kinase-inactive K508M mutant or empty vector, suggesting each has this capacity. To control for variance created by differential transgene expression within one experiment, we repeated the experiment shown in Fig. 3B five times. Again, all six mutants inhibited the growth of RCS chondrocytes with Y373C, K650M and K650E-FGFR3 being the strongest inhibitors (Fig. 3C).

When compared with cells tranfected with empty vector or with the kinase-inactive K508M mutant, the wild-type FGFR3 also inhibited RCS growth (Fig. 3B, C). It is likely that ectopic expression of wild-type FGFR3 leads to its activation and subsequent RCS growth arrest, similar to that described for B9 cells [32].

### STAT1 and ERK activation by FGFR3 mutants

Our study compares six different FGFR3 mutants associated with skeletal dysplasias for their activation of the STAT1 and ERK MAP kinase pathways. According to our previous study compiling the clinical data of 591 patients, the N540K, G380R, R248C, Y373C, K650M and K650E-FGFR3 mutants used here account for 92.9% of cases [1]. Therefore, by clinical prevalence criteria, the mutants studied represent a majority of known FGFR3-related dysplasia cases.

Our results demonstrate that activation of STAT1 is limited mostly to the K650M and K650E-FGFR3 in the experimental settings used here (Figs 1–3). Although it is possible that the other tested mutants also activate STAT1, this activity is undetectable or much lower when compared to K650M and K650E-FGFR3 (Fig. 2). In fact, we show that wild-type, N540K, G380R, R248C and Y373C-FGFR3 activate STAT1 poorly despite the experimental conditions used here, i.e. kinase reaction where both FGFR3 and STAT1 are used in excess as well as *in vitro* cell experiments with overexpressed FGFR3. We thus conclude that, among the FGFR3 mutants associated with skeletal dysplasia, only K650M and K650E possess an unique substrate specificity towards STAT1, possibly through intracellular signaling mechanisms [22].

**Figure 4:**
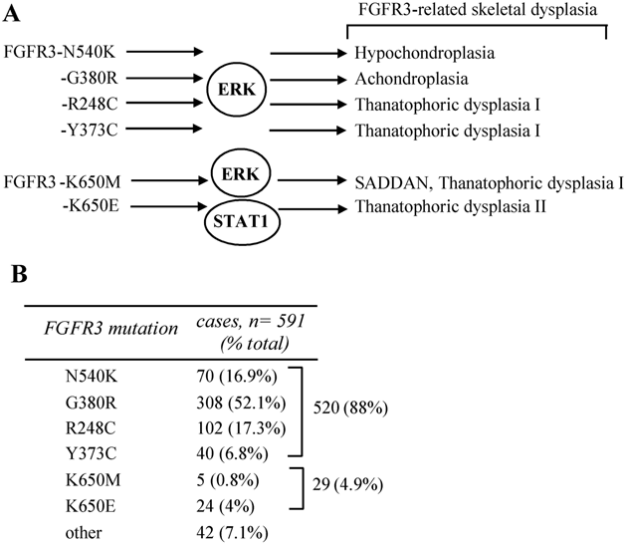
STAT1 and ERK1/2 activation by FGFR3 mutants. (A) The N540K, G380R, R248C, Y373C, K650M and K650E-FGFR3 mutants used in this study all cause FGFR3-skeletal dysplasias and signal through ERK MAP kinase in contrast to STAT1, that is activated mostly by the K650M and K650E-FGFR3 mutants. (B) According to the study compiling the clinical data of 591 patients suffering from FGFR3-related skeletal dysplasia [1], the STAT1-activating K650M and K650E account for as little as 4.9% of cases. It is therefore unlikely that activation of STAT1 plays a central role in FGFR3-related skeletal dysplasias as currently believed, but rather represents a signaling feature unique to small subset of patients carrying the K650M and K650E mutations.

Taken together, we demonstrate that only K650M and K650E-FGFR3 mutants represent the only significant activators of STAT1 among the mutants studied here (Figs 1–3). As K650M and K650E mutations account only for a minority of the FGFR3-related dysplasia cases (4.9%) [1], activation of STAT1 does not cause the disease in majority of the cases (Fig. 4). Other pathways should therefore be considered as prominent in the pathological FGFR3 signaling in skeletal dysplasias. This may be ERK MAP kinase pathway, which is a candidate for the FGFR3-mediated chondrocyte growth arrest [9], [17], [18], is more or less uniformly induced by N540K, G380R, R248C, Y373C, K650M and K650E-FGFR3 mutants used here (Figs 2, 3), and activation of this pathway largely replicates the skeletal phenotype of FGFR3 mutations [16], [19].

### STAT5 activation by FGFR3 mutants in RCS chondrocytes

**Figure 5:**
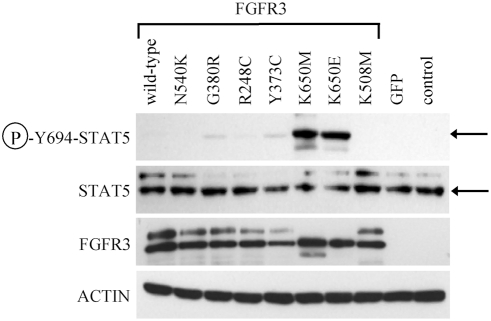
STAT5 activation by FGFR3 mutants. RCS chondrocytes transfected with vectors expressing wild-type FGFR3, activating FGFR3 mutants (N540K, G380R, R248C, Y373C, K650M and K650E), and kinase-inactive mutant K508M were grown for 24 hours and analyzed for the indicated molecules by western blotting. Note the significant STAT5(Y694) phosphorylation induced by K650M and K650E-FGFR3. The membrane used for P-STAT5-Y694 detection was reprobed with antibody recognizing STAT5 regardless of its phosphorylation. Arrow indicates P-STAT5(Y694) or STAT5 signal. FGFR3 and ACTIN western blottings serve as loading controls. Cells transfected by GFP vector serve as negative control for transfection.

In addition to STAT1, STAT5 was also found activated by FGFR3 mutants in cartilage *in vivo* [12], [13]. We therefore tested the ability of N540K, G380R, R248C, Y373C, K650E and K650M-FGFR3 mutants to activate STAT5 in RCS chondrocytes. Cells were transfected with N540K, G380R, R248C, Y373C, K650M and K650E-FGFR3 mutants and analyzed for activatory STAT5(Y694) phosphorylation 24 hours later. Figure 5 shows that only the K650M and K650E mutants caused significant STAT5(Y694) phosphorylation in RCS chondrocytes. This phenotype was obtained with two different P-STAT5(Y694) antibodies thus ruling-out the cross-reactivity with STAT1. Identical results were obtained with HeLa and 293T cells (not shown). Our data thus suggest that signaling of FGFR3 mutants towards STAT5 is similar to STAT1, i.e. only the K650M and K650E-FGFR3 are significant activators of STAT5.

## Materials and Methods

### Cell culture, Western blotting (WB) and immunoprecipitation

Rat chondrosarcoma (RCS) chondrocytes were obtained from C. Basilico; CHO, HeLa and 293T cells were obtained from the Tissue Culture Core at Cedars-Sinai Medical Center, Los Angeles, CA. All cell lines were propagated in DMEM media (Gibco, Gaithersburg, MD), containing 10% FBS (Atlanta Biological, Nordcross, GA) and antibiotics, with exception for CHO cells which were maintained in Opti-MEM media (Gibco). For WB or immunoprecipitation, cells were lysed in buffer containing 50 mM Tris-HCl pH 7.4, 150 mM NaCl, 0.5% NP-40, 1 mM EDTA, 25 mM NaF, 0.1 mM DTT, 1 µg/ml leupeptin, 10 µg/ml soybean trypsin inhibitor, 1 mM PMSF, 8 mM β-glycerolphosphate, 10 mM Na_3_VO_4_ and 1 µg/ml aprotinin. Lysates were resolved by SDS-PAGE, transferred onto a PVDF membrane and visualized by luminescence (Amersham, Piscataway, NJ). The following antibodies were used: ACTIN, FGFR1, FGFR2, FGFR3 and FGFR4 (Santa Cruz Biotechnology, Santa Cruz, CA); ERK1/2, P-ERK1/2^T202/Y204^, STAT1, STAT5, P-STAT1^Y701^, and P-STAT5^Y694^ (Cell Signaling, Beverly, MA); P-STAT5^Y694^ (BD Transduction Laboratories, San Diego, CA). To quantify the WB signal, the integrated optical density (I.O.D) of a given band was determined using Scion Image software (Scion Corporation, Frederick, MA).

### FGFR3 kinase assays

FGFR3 kinase assays were carried out as described before [11], [23]. Briefly, kinase reactions were performed for 30 minutes at 30°C in 50 µl of kinase buffer (60 mM Hepes-NaOH pH 7.5, 3 mM MgCl_2_, 3 mM MnCl_2_, 3 µM Na_3_VO_4_) supplemented with 2.5 µg polyethylene glycol, 10 µM ATP and 300 ng of recombinant human STAT1 (Active Motif, Carlsbad, CA) as a substrate. The recombinant FGFR3 intracellular tyrosine kinase domain (E322-T806; Cell Signaling) was used at 300 ng per reaction. For the kinase assays utilizing full-length FGFR3, vectors carrying C-terminally FLAG-tagged wild-type FGFR3 as well as N540K, G380R, R248C, Y373C, K650M and K650E-FGFR3 mutants were transfected into CHO cells. Forty eight hours after transfection, cells were treated with 20 ng/ml of FGF2 (R&D Systems, Minneapolis, MN) for 15 minutes and FGFR3 was immunoprecipitated from 800 µg of cell lysate protein using 4 µg anti-FLAG antibody (Sigma-Aldrich, St. Louis, MO). Cells transfected with plasmid encoding green fluorescent protein (GFP; pCCEY) serve as a negative control for the immunoprecipitation. Immunocomplexes were washed two times with kinase buffer and kinase reactions were carried-out as described above.

### Vectors and cell transfection

The pRK7 vectors carrying FLAG-tagged wild-type, K650E and K508M-FGFR3 constructs were described previously [23]. FLAG-tagged N540K and K650M-FGFR3 were generated in the same manner. To produce the FLAG-tagged G380R, R248C, and Y373C-FGFR3 constructs, FLAG-tagged wild-type FGFR3 was subjected to site-directed mutagenesis using the Quick Change II Site-Directed Mutagenesis Kit (Stratagene, La Jolla, CA) according to the manufacturer's protocol. The following mutagenic primers were used (forward, reverse): R248C 5′-GACGTGCTGGAGTGCTCCCCGCACC-3′, 5′-GGTGCGGGGAGCACTCCAGCACGTC-3′; Y373C (5′-CGAGGCGGGCAGTGTGTGTGCAGGCAT-3′, 5′-ATGCCTGCACACACACTGCCCGCCTCG-3′; and G380R 5′-GCATCCTCAGCTACAGGGTGGGCTTCTTC-3′, 5′-GAAGAAGCCCACCCTGTAGCTGAGGATGC-3′. The vector containing no insert was pcDNA3. Before transfection, the RCS extracellular matrix was degraded by 0.3% bacterial collagenase (type II; Invitrogen, Carlsbad, CA). Cells were transfected with FuGENE6 (Roche Diagnostics, Penzberg, Germany) according to the manufacturer's protocol, the plasmid (µg) versus FuGENE6 (µl) ratio was 1:3. For WB experiments, 2×10^5^ cells grown in 6-well tissue culture plates (Costar, Cambridge, MA) was transfected with 2.8 µg of plasmid. For RCS growth assay experiments, 1×10^4^ cells seeded in 24-well tissue culture plates (Costar) was transfected with 1.3 µg of plasmid, cultivated for 72 hours and counted.
